# Cognitive function in adult NF1 patients without other cognitive disorders: a cross-sectional study

**DOI:** 10.1186/s13023-026-04456-6

**Published:** 2026-07-03

**Authors:** Daniel Muñoz, Juan Luis Becerra, Elisabeth Castellanos, Victoria Aran, Emilio Amilibia, Montse Pauta, Belén García, Andrea Ros, Ignacio Blanco, Mercè Jodar

**Affiliations:** 1 Clinical and Health Psychology Department, Universitat Autònoma de Barcelona (UAB), Bellaterra, Spain; 2 Clinical Genomics Group, CARE Program, Institut d’investigació Germans Trias i Pujol (IGTP), Badalona, Spain; 3 CSUR of Phacomatoses, Clinical Genetics Department, Hospital Universitario Germans Trias I Pujol, Badalona, Spain; 4 Department of Surgery, Universitat Autònoma de Barcelona (UAB), Bellaterra, Spain; 5 Neurology Department, Hospital Universitari Parc Taulí, Sabadell, Spain; 6 Institut d’Investigació i Innovació Parc Taulí (I3PT), Centro de Investigación Biomédica en Red – Salud Mental (CIBERSAM), Sabadell, Spain

**Keywords:** Neurofibromatosis type 1, Adults, Neuropsychological assessment, Executive function, Attention, Depression, Disease severity

## Abstract

**Background:**

Neurofibromatosis type 1 (NF1) is associated with cognitive impairments affecting attention, executive function, memory, visuospatial abilities, and processing speed, which are well described in children and adolescents and may interfere with daily functioning. In contrast, cognitive functioning in adults with NF1 remains less clearly defined, particularly in individuals without previous neurological or psychological conditions affecting cognitive functioning that may confound neuropsychological performance. This cross-sectional study aimed to characterize cognitive performance across multiple domains in adults with NF1 and to examine the relationship between cognitive performance and psychological and clinical variables.

**Results:**

Eighty-seven adults with NF1 and no intellectual disability or previously diagnosed cognitive or psychiatric disorders underwent standardized neuropsychological assessment across seven cognitive domains commonly reported as affected in NF1, particularly in pediatric populations: visual memory, verbal memory, executive function, attention, visuospatial ability, working memory, and visuomotor speed. Participants also completed questionnaires assessing sociodemographic and psychological variables. Cognitive performance was standardized using age-adjusted normative Z-scores. Adults with NF1 performed below the normative mean across several cognitive domains relative to age-adjusted Spanish reference data. Executive function showed clinically significant impairment (z = −2.267), whereas attention showed low-average performance (z = −1.171). Although attention was associated with skin severity and visuospatial ability with depressive symptoms (*p* < 0.05), these associations were not significant after correction for multiple comparisons.

**Conclusions:**

These findings suggest a consistent pattern of cognitive underperformance in adults with NF1 without previous neurological or psychological conditions affecting cognitive functioning, supporting the presence of a specific cognitive vulnerability associated with the condition. Although some clinical factors showed associations with specific cognitive domains, these relations were small and did not remain significant after correction for multiple comparisons. These results highlight the importance of routine cognitive assessment and targeted cognitive and psychosocial support strategies, including their integration into genetic counseling, to improve clinical follow-up and patient care.

**Supplementary Information:**

The online version contains supplementary material available at https://doi.org/10.1186/s13023-026-04456-6.

## Background

Neurofibromatosis type 1 (NF1) is an autosomal dominant genetic disorder caused by pathogenic variants in the *NF1* gene, with a birth prevalence estimated between 1:2,000 and 1:3,000 individuals, although recent studies suggest it may be as high as 1:1,286 due to mosaicism and mild undiagnosed cases [1, 2]. Clinically, NF1 is characterized by café-au-lait macules, skin-fold freckling, Lisch nodules, scoliosis, and benign peripheral nerve tumors (neurofibromas), including plexiform neurofibromas with risk of malignant transformation (MPNST) [1, 3–5].

NF1 is associated with cognitive impairments, largely due to reduced or absent neurofibromin, the protein encoded by the NF1 gene. Neurofibromin normally downregulates the RAS pathway; its loss leads to RAS hyperactivation, which contributes to neurological deficits. Research in human and animal models link this NF1 typical dysregulation to increased inhibitory signaling via γ-aminobutyric acid (GABA) in brain regions critical for learning, memory, and executive functions [6–10].

Research on NF1 has largely focused on pediatric populations, showing cognitive deficits in executive function, visuospatial skills, attention, processing speed, working memory, language, and motor abilities, with clear impacts on academic and daily life [6, 11–14]. Social difficulties, including impaired non-literal language, theory of mind, and emotion recognition, also affect peer relationships [12, 15–17]. In contrast, studies in adults are scarce but report lower general intelligence, a higher prevalence of intellectual disability (6–7%), and neurocognitive alterations in memory, attention, visuospatial processing, procedural learning, social cognition and motor control [6, 14, 18–20]. Similar patterns are seen in elderly NF1 patients, though generally less pronounced [21].

Despite these findings, research on cognitive functioning in adults and older individuals with NF1 remains limited and presents several methodological challenges. Samples tend to be small and heterogeneous, with a wide variety of assessment protocols and cognitive outcomes are often influenced by comorbidities more common in adulthood, limiting comparability and generalizability [6, 14, 18, 20, 22, 23]. Moreover, few studies specifically examine adults without previous cognitive or intellectual disorders. These limitations highlight the need for further research to better characterize cognitive domains in adults with NF1, particularly in those without cognitive comorbidities [14, 20, 22].

Cognitive deficits in NF1 may also be influenced by psychological factors such as pain, social functioning, emotional problems (depression and anxiety), quality of life (QoL), and body image (BI). Recurrent pain can interfere with cognitive performance and daily functioning [24–27], while social difficulties may exacerbate pain perception and are associated with emotional distress [28]. Moreover, children with NF1 show reduced attention to faces and delayed neural responses to sound changes, patterns that resemble those observed in atypical autism [29]. In this population, higher levels of depression and anxiety have been associated with poorer cognitive outcomes, particularly in executive functioning, which is closely tied to emotional regulation. When this regulation is impaired, it contributes to reduced QoL, especially in social contexts [12, 30, 31]. Finally, body image is another important domain affected in NF1. In adults, increased disease visibility often correlates with greater severity and has been associated with cognitive impairments. Additionally, dissatisfaction with body image is linked to emotional distress, which can further impair cognitive functioning [30, 32].

In accord with this, this research aims to describe the cognitive function of the adults NF1, without previous neurological or psychological conditions affecting cognitive functioning, expecting that they will exhibit a lower performance in the assessed cognitive domains compared to the general population. Additionally, the study evaluates associated psychological outcomes, including pain, body image, anxiety, depression, quality of life and skin involvement, with the expectation that these variables may be associated with reduced cognitive performance.

## Methods

A sample of 87 patients with NF1 who attended the Hospital Germans Trias i Pujol phacomatoses unit, as a national reference center for this type of pathology in the province of Barcelona (CSUR), were included in the study. The study was approved by the Clinical Research Ethics Committee (CEIC) of Hospital Universitari Germans Trias i Pujol (Ref. PI-20–184) and was conducted in accordance with the latest version of the ethical principles of the Declaration of Helsinki. NF1 diagnosis was evaluated following the revised guidelines proposed by Legius et al. 2021 [1]. All participants attended for their scheduled follow-up. Before the assessment, they signed informed consent, and all questionnaires and cognitive tests were administered by a trained psychologist.

The study followed a cross-sectional design and adhered to the Strengthening the Reporting of Observational Studies in Epidemiology (STROBE) guidelines [33].

The inclusion criteria consisted of adults diagnosed with NF1 without previous neurological or psychological conditions affecting cognitive functioning. Patients with a diagnosis of hydrocephalus, moyamoya vasculopathy, epilepsy, Chiari malformation or intellectual disability were not included. Additionally, participants with a prior clinical diagnosis of major depressive disorder or generalized anxiety disorder were not eligible for inclusion in the study [27, 28, 34–36]. To minimize selection bias, all eligible patients attending the unit between January 2023 and May 2024 who consented to participate were consecutively enrolled, resulting in an initial sample of 102 participants. All eligible participants were administered the neuropsychological tests and clinical scales included in the study protocol.

In addition, participants presenting elevated depressive symptomatology according to the Hospital Anxiety and Depression Scale (HADS-Depression subscale score > 8) were excluded from the final sample. Application of this criterion reduced the sample from 102 to 87 participants. Preliminary analyses conducted during the study indicated that higher depressive symptom scores were associated with lower performance in the Global Cognitive Index (GCI), suggesting that depressive symptoms could confound the interpretation of NF1-related cognitive functioning. Therefore, these criteria were applied to better characterize cognitive performance associated specifically with NF1 rather than secondary psychological distress. This also served to control potential confounding factors and enhance the robustness of the sample.

**Fig. 1:**
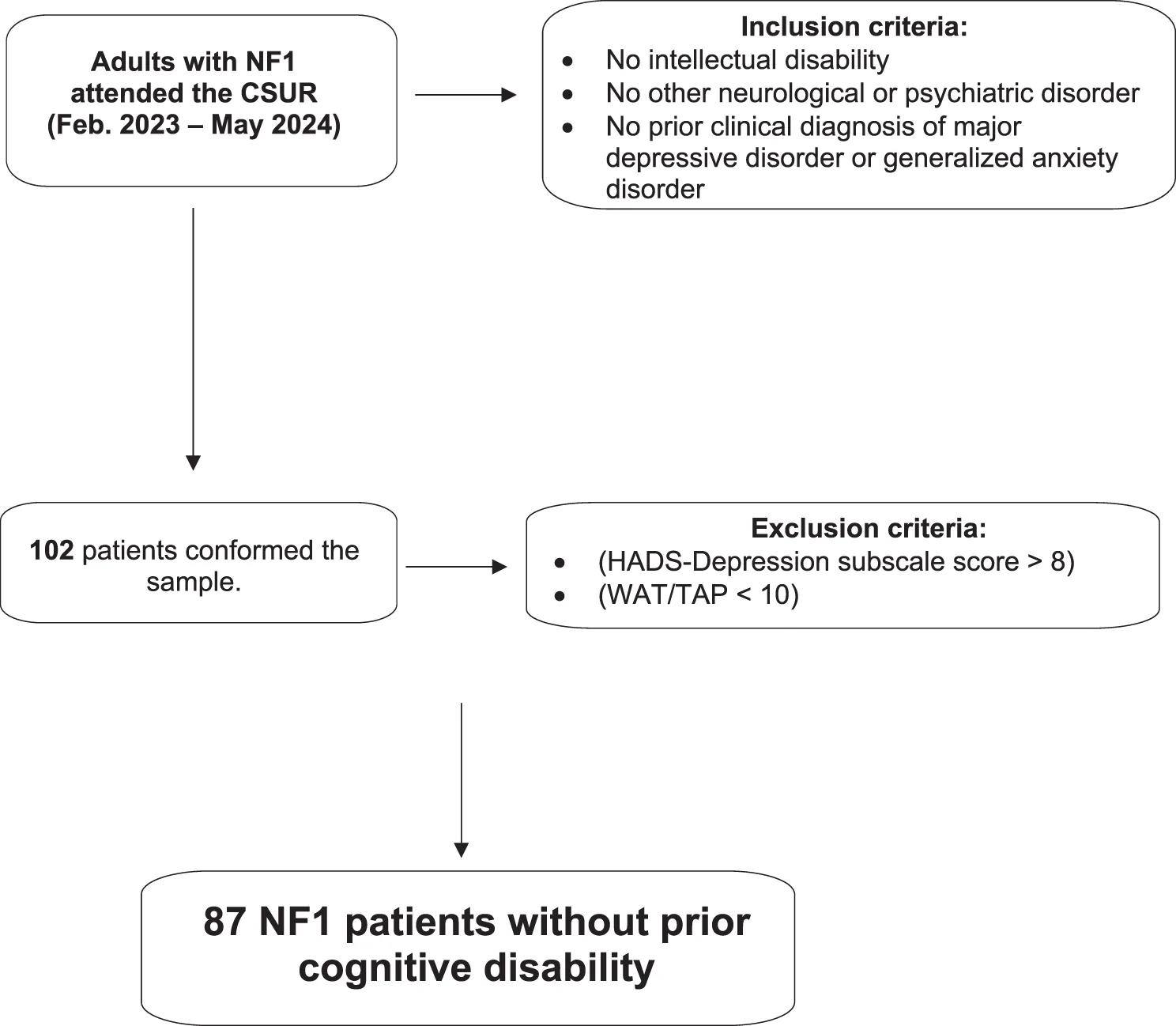
Participant selection flow diagram

Finally, premorbid intellectual functioning was estimated using the National Adult reading test (NART). Participants scoring below 10 points (NART < 10) were excluded [37, 38]. However, no additional participants met this exclusion criterion. Screening procedures and eligibility criteria were verified by trained psychologists from the phacomatoses reference unit (Fig. 1).

The cognitive function of NF1 patients was assessed using standardized neuropsychological tests, with each cognitive domain represented by a single primary measure: Logical Memory for verbal memory, Visual Reproduction for visual memory, Digit Span Forward for attention, Digit Span Backward for working memory, Symbol Search for processing speed, Judgment of Line Orientation for visuospatial functioning, and the Trail Making Test for executive functioning.

## Assessment

**Sociodemographic data:** Patients provided their age, education, marital status, employment status, psychological treatment history, and social support.

The **Spanish Body Image Scale (S-BIS)** is a 10-item measure assessing affective, behavioral, and cognitive aspects of body image in cancer patients. Each item is rated on a 4-point scale, with total scores ranging from 0 to 30, Higher scores reflect greater body image concerns [39]. The modified version of the S-BIS was used in this study, adapted from the original S-BIS to be used in NF1 patients. Items 8 and 10 were removed, as they refer to feeling the body incomplete or having scars, which are not typical concerns in NF1. The modified version retained the same psychometric properties (validity and reliability) as the original [40].

**Skin severity index** was assessed using a 3-point clinical developed according to our hospital’s NF1 clinical guidelines. [1]. These grades were defined as follows:

- Grade 1 (mild): presence of some clinical features, such as a café au lait spots, and small number of neurofibromas.
- Grade 2 (moderate): There is no significant compromise to the patient’s health; presence of café au lait spots, and a moderate number of cutaneous and/or subcutaneous neurofibromas.
- Grade 3 (severe): presence of plexiform neurofibromas and/or a high number of cutaneous and subcutaneous neurofibromas throughout the body. These skin manifestations affected the patient’s health.

The **Hospital Anxiety and Depression Scale (HADS**) is a 14-item self-report questionnaire that screens for anxiety and depression in non-psychiatric medical outpatient settings. It includes two 7-item subscales (anxiety and depression), each ranging from 0 to 21. Symptoms are assessed over the past week, with scores of 0–7 indicating no clinical relevance, 8–10 suggesting the need for further evaluation, and 11–21 signaling probable clinical anxiety or depression. Internal consistency of the HADS was assessed in the present sample. Cronbach’s alpha indicated good reliability for the Anxiety subscale (α = 0.83), whereas the Depression subscale showed lower internal consistency (α = 0.61) (Additional File 1) [41, 42].

The **Pain Numerical Rating Scale (PNRS)** was used to rate patients’ average pain over the past week using a scale from 0 (no pain) to 10 (extremely painful). Patients indicate their pain level by marking a number on a visual scale [43]

The **EuroQol Quality of Life Scale (EQ-5D)** was used to assess the QoL across five dimensions: mobility, personal care, daily activities, pain/discomfort, and anxiety/depression, each rated at three severity levels [44]. It also includes the EQ-Visual Analogue Scale (VAS), a visual analog scale from 0 (worst health) to 100 (best health) for overall health assessment [45].

## Neuropsychological tests

- The **National Adult reading test (NART)** was used to assess general intelligence by estimating premorbid intelligence in Spanish speakers. It involves reading aloud 30 uncommon Spanish words that are printed in bold, uppercase black letters, without accent marks. Patients are asked to correctly stress the syllables of each word, without needing to consider the word’s meaning. The final score reflects the number of correctly stressed words [46].
- **Logical Memory I and II from Wechsler Memory Scale (WMS-IV)**. WMS-IV assesses verbal memory in individuals aged 16–89 through two batteries: the Adult Battery (16–69 years) and the Older Adult Battery (65–89 years). This study used the Logical Memory subtest to evaluate short- and long-term verbal memory, measuring immediate narrative recall (Logical Memory I) and delayed recall after 30 minutes with recognition cues (Logical Memory II) [47].
- **Visual Reproduction I and II from Wechsler Memory Scale (WMS-IV)** was used to assess visual memory through immediate (Visual Reproduction I) and delayed recall 30 minutes later of figures with visual information (Visual Reproduction II) [47].
- **Digit Span from Wechsler Adult Intelligence Scale (WAIS IV).** It was used to measure attention and executive function (working memory). The task involves repeating number sequences forward and backwards. Only the forward and reverse digit span tasks were used in this study. [48].
- **Symbols from Wechsler Adult Intelligence Scale (WAIS IV)** were used to evaluate visuomotor speed. The symbols task require participants to copy symbols matched with numbers. [48].
- The **Trail Making Test (TMT)** assessed executive function (Cognitive Flexibility) through two parts: A (connecting numbers in ascending order to measure sustained attention and hand-eye coordination speed) and B (alternating numbers and letters to evaluate cognitive flexibility and mental flexibility). [49]. To obtain independent measures of processing speed, the difference in time taken to complete TMT-B and the time to complete TMT-A was calculated. Results were analyzed using normative reference data expressed as T-scores derived from English-language samples, which were selected due to their greater sensitivity for detecting executive dysfunction. [50]
- The **Color Trail Making Test (CTT)**, an adaptation of the TMT, assesses executive function while minimizing educational biases. It includes two parts: CTT-1 (connecting numbered circles sequentially) and CTT-2 (alternating between yellow and pink circles) [51]. Results were analyzed using normative data from a Spanish sample. [52].
- The **Judgment of Line Orientation (JLO)** test evaluated visuospatial ability. It measures the ability to assess spatial relationships between lines presented to the subject, who must match a given line to a model based on its angle of inclination. The full test consists of 30 items and has two alternative forms (H and V) [53]. Results were analyzed with the normative data available for Spanish sample. [54].

## Statistical analysis

Statistical analyses were conducted using R version 4.2.2, including descriptive and bivariate analyses. The Shapiro–Wilk test assessed data distribution (Additional File 2). Quantitative variables were reported as means and standard deviations, while qualitative variables were presented as frequencies and percentages [55]. Raw scores from the administered cognitive tests were converted into scaled scores (Pe; *M* = 10, SD = 3) according to age-adjusted norms from standardized Spanish population samples, and these scaled scores were then transformed into Z-scores [(x - population mean)/ population SD]. The only exception was the Trail Making Test (TMT), for which standardized Z-scores were obtained from normative T-scores based on English-language reference data. These norms were selected because they provide greater sensitivity for detecting executive dysfunction and the TMT is considered largely language-independent, allowing valid cross-cultural application [50]. Interpretation of performance relative to the normative population was based on these test-specific Spanish reference datasets [46–48, 54]. The GCI was calculated as the mean of standardized Z-scores across the neuropsychological measures included in the assessment and was conceived as a descriptive composite indicator to summarize global cognitive performance across heterogeneous cognitive measures and did not replace domain-specific analyses [56]. To illustrate the cognitive functions of the NF1 patients, domains and test results were represented using bar plots. Normal performance was defined within two standard deviations above or below the mean (±2) [57].

Spearman correlations were conducted between the GCI, cognitive domains, and other variables, including sociodemographic data, HADS, S-BIS modified, Skin Involvement Index, EuroQoL, and PNRS to examine relationships and control for potential confounding factors. Spearman correlation coefficients (ρ) were interpreted as effect size estimates following conventional thresholds (ρ ≈ 0.10 small, ≈ 0.30 moderate, ≈ 0.50 large), and 95% confidence intervals were calculated to improve interpretability of associations [58]. Given the large number of correlations performed, false discovery rate (FDR) correction using the Benjamini–Hochberg procedure was applied to reduce the risk of Type I error due to multiple comparisons [59].

## Results

The final sample consisted of 87 patients with NF1 aged in a range between 16 years to 74 years. Participants were, altogether, single (72%), employed (56%), reported having social support (70%) and reported not having received psychological counseling (79%). On average, patients had 12 years of education, their skin severity index was low (1.94) and the average age was 35 years. Overall, they reported no depression nor anxiety (2.92/ 6.48), good QoL (0.77/75.26), low body image-related problems (5.07), and low level of feeling pain (2.55) (Table 1).

**Table 1:** Correlations between sociodemographic/clinical variables and Global cognitive Index in adults with NF1

Variables	Categories/ Ranges	Global Cognitive Index (n = 87)				
		M (SD)/ N (%)	Spearman (ρ)	95% CI (ρ)	p-value	p (FDR–BH)
Age	16 - 74	34.89 (14.85)	0.150	[−0.063 - 0.349]	0.166	0.752
Marital Status	Single	63 (72.41)	-	-	-	-
	Married	23 (26.44)	−0.061	[−0.268 - 0.151]	0.573	0.953
	Divorced	1 (1.15)	−0.146	[−0.346 - 0.067]	0.177	0.752
	Widowed	0 (0)	-	-	-	-
Education	0 - 17	12.28 (2.85)	0.308	[0.104 - 0.487]	0.004**	0.067
Employment	Unemployed	28 (32.18)	-	-	-	-
	Employed	49 (56.32)	0.014	[−0.197 - 0.224]	0.953	0.953
	Paid leave	4 (4.60)	−0.037	[−0.246 - 0.175]	0.733	0.953
	Retired	6 (6.90)	−0.038	[−0.247 - 0.174]	0.855	0.953
Psychological Counseling	No	69 (79.31)	-	-	-	-
	Yes	18 (20.69)	0.054	[−0.159 - 0.261]	0.625	0.953
Social support	No	26 (29.88)	-	-	-	-
	Yes	61 (70.11)	0.090	[−0.121 - 0.297]	0.397	0.923
Skin Involvement Index	1 - 3	1.94 (0.91)	0.074	[−0.139 - 0.280]	0.497	0.953
	Grade 1 (mild)	38 (43.68)	-	-	-	-
	Grade 2 (moderate)	16 (18.39)	−0.133	[−0.335 - 0.079]	0.218	0.752
	Grade 3 (severe)	33 (37.93)	0.124	[−0.090 - 0.325]	0.250	0.752
HADS	DEP (0 - 21)	2.92 (2.34)	−0.178	[−0.374 - 0.034]	0.100	0.752
	ANS (0 - 21)	6.48 (3.89)	0.026	[−0.186 - 0.235]	0.812	0.953
EuroQoL	0.00 - 1.00	0.77 (0.26)	0.008	[−0.203 - 0.218]	0.944	0.953
EuroQol Visual Scale	0 - 100	75.26 (19.75)	−0.091	[−0.298 - 0.125]	0.410	0.923
S-BIS modified	0 - 24	5.07 (6.27)	0.030	[−0.182 - 0.239]	0.785	0.953
PNRS	0 - 10	2.55 (3.04)	0.013	[−0.199 - 0.224]	0.904	0.953

*N* = number of participants; % = percentage; *M* = mean; SD = standard deviation; ρ = Spearman’s correlation coefficient; CI = confidence interval; p significance levels: <0.05 (*), <0.01 (**), <0.001 (***), FDR–BH = false discovery rate corrected *p*-value (Benjamini–Hochberg). HADS = Hospital Anxiety and Depression Scale; S-BIS modified = Spanish Body Image Scale (modified); PNRS = Pain Numerical Rating Scale. The first category of each categorical variable was used as the reference group

**Fig. 2:**
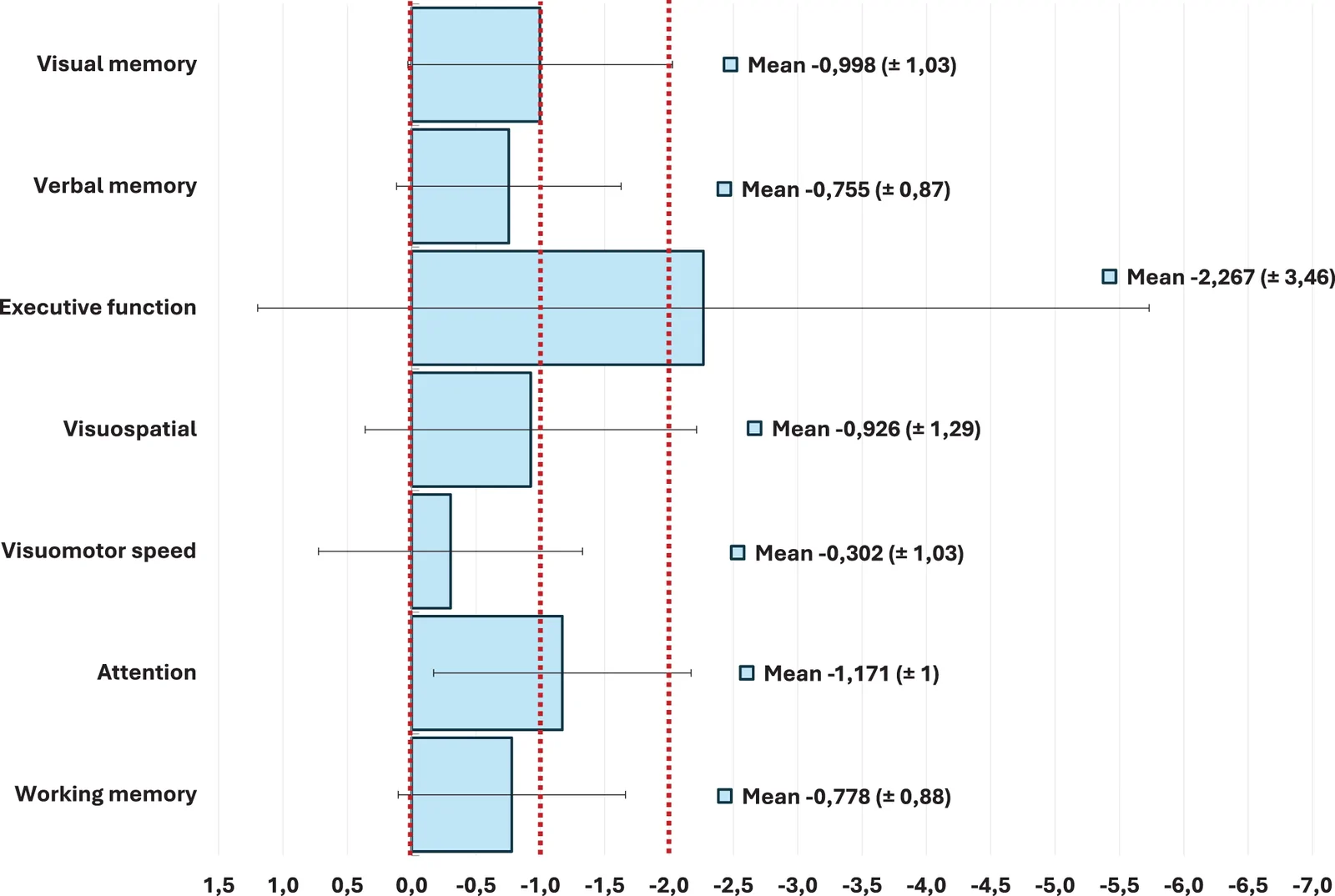
Cognitive performance in adult neurofibromatosis type 1 patients: results of the seven cognitive dimensions assessed. *Bars represent the mean z-scores of participants across cognitive domains relative to age-adjusted normative data. Error bars represent ±1 standard deviation of the sample. Red dashed lines indicate the normative mean (0) and thresholds at − 1 and −2 standard deviations

Seven cognitive dimensions were assessed: visual memory, verbal memory, executive function, visuospatial ability, visuomotor speed, attention and working memory. Of the seven evaluated domains, only executive function showed clinically significant impairment (z = −2.267; SD = 3.46). The remaining domains, although within the normal range, scored below the population mean. Notably, attention showed a relatively lower performance compared to normative expectations (z = −1.171; SD = 1.00), falling within the low-average range and approaching the threshold typically considered clinically relevant. These findings suggest subtle weaknesses in attention alongside clinically significant impairment in executive function (Fig. 2).

**Fig. 3:**
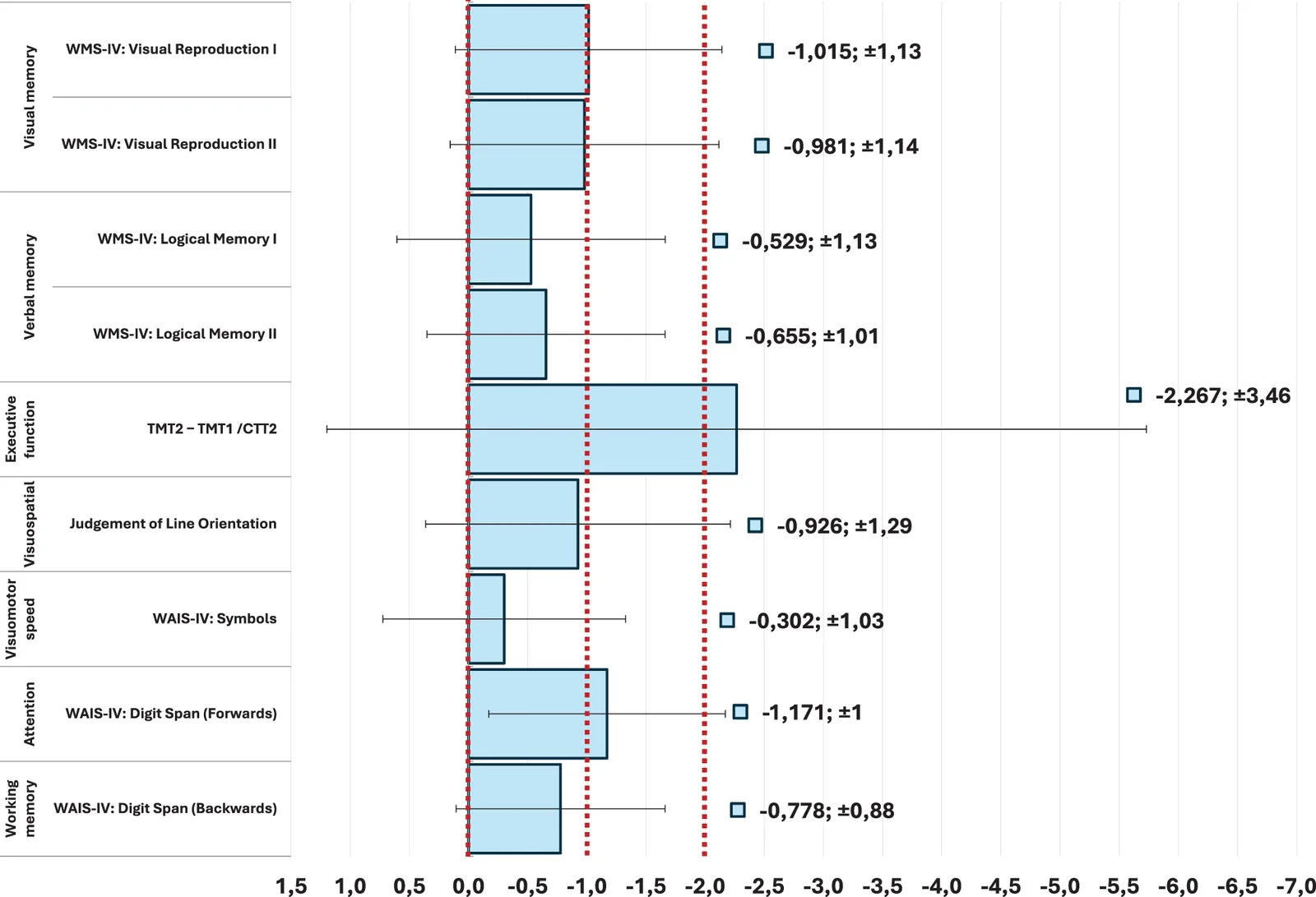
Cognitive performance in adult neurofibromatosis type 1 patients: results of each cognitive task assessed *Bars represent the mean z-scores of participants across cognitive domains relative to age-adjusted normative data. Error bars represent ±1 standard deviation of the sample. Red dashed lines indicate the normative mean (0) and thresholds at − 1 and −2 standard deviations

To further illustrate performance across cognitive domains, a bar graph was presented displaying the mean z-scores obtained by the sample for each cognitive test, linking each score to the specific cognitive function assessed. Although most scores remained within the normal range, visual memory performance approached one standard deviation below the normative mean, with short-term visual memory (z = −1.015; SD = 1.13) falling within the low-average range. This pattern suggests relatively lower performance compared to population expectations, while remaining within normative limits (Fig. 3).

The analysis of correlations between the GCI and the clinical variables assessed in adults with NF1 revealed no significant associations. Years of education showed a positive correlation with the Global Cognitive Index at the uncorrected level (ρ = 0.314, *p* = 0.003); however, this association did not remain significant after correction for multiple comparisons (Table 1).

When analyzing specific cognitive domains, distinct associations were observed with sociodemographic factors across several of the seven domains studied. Years of education showed a moderate positive correlation with visual memory (ρ = 0.342, 95% CI [0.142–0.515], *p* < 0.001), which remained significant after correction for multiple comparisons. No clinical variables were significantly associated with visual memory performance. For the verbal memory domain, no significant associations were observed with sociodemographic or clinical variables. For the executive function domain, years of education (ρ = 0.267, 95% CI [0.060–0.452], *p* = 0.012) and paid leave status (ρ = 0.263, 95% CI [0.056–0.449], *p* = 0.014) showed positive correlations at the uncorrected level; however, these associations did not remain significant after correction for multiple comparisons. No clinical variables were significantly associated with executive function performance (Table 2, 3, 4).

**Table 2:** Correlations between sociodemographic/clinical variables and visual memory Z-scores in adults with NF1

Variables	Categories/ Ranges	Visual memory			
		Spearman (ρ)	95% CI (ρ)	*p*-value	p (FDR–BH)
Age	16 - 74	0.058	[−0.155 - 0.265]	0.596	0.823
Marital Status	Single	-	-	-	-
	Married	−0.110	[−0.313 - 0.103]	0.312	0.804
	Divorced	−0.176	[−0.373 - 0.036]	0.103	0.615
Education	0 - 17	0.342	[0.142 - 0.515]	<0.001***	0.021*
Employment	Unemployed	-	-	-	-
	Employed	0.051	[−0.162 - 0.259]	0.640	0.823
	Paid leave	−0.130	[−0.332 - 0.083]	0.229	0.804
	Retired	−0.066	[−0.273 - 0.147]	0.544	0.823
Psychological Counseling	No	-	-	-	-
	Yes	0.037	[−0.175 - 0.246]	0.735	0.845
Social support	No	-	-	-	-
	Yes	0.035	[−0.177 - 0.243]	0.751	0.845
Skin Involvement Index*	1 - 3	0.088	[−0.125 - 0.293]	0.416	0.823
HADS	DEP (0 - 21)	−0.207	[−0.400 - 0.004]	0.055	0.492
	ANS (0 - 21)	−0.003	[−0.214 - 0.208]	0.977	0.977
EuroQoL	0.00 - 1.00	0.112	[−0.101 - 0.315]	0.301	0.804
EuroQol Visual Scale	0 - 100	−0.062	[−0.272 - 0.153]	0.572	0.823
S-BIS modified	0 - 24	−0.027	[−0.236 - 0.185]	0.804	0.851
PNRS	0 - 10	−0.055	[−0.264 - 0.159]	0.615	0.823

ρ = Spearman’s correlation coefficient; CI = confidence interval; p significance levels: <0.05 (*), <0.01 (**), <0.001 (***), FDR–BH = false discovery rate corrected *p*-value (Benjamini–Hochberg). HADS = Hospital Anxiety and Depression Scale; S-BIS modified = Spanish Body Image Scale (modified); PNRS = Pain Numerical Rating Scale. The first category of each categorical variable was used as the reference group. *Skin involvement index analyzed as a quantitative variable for correlation

**Table 3:** Correlations between sociodemographic/clinical variables and verbal memory Z-scores in adults with NF1

Variables	Categories/ Ranges	Verbal memory			
		Spearman (ρ)	95% CI (ρ)	*p*-value	p (FDR–BH)
Age	16 - 74	0.095	[−0.118 - 0.300]	0.380	0.712
Marital Status	Single	-	-	-	-
	Married	−0.118	[−0.321 - 0.095]	0.274	0.699
	Divorced	−0.181	[−0.377 - 0.031]	0.094	0.438
Education	0 - 17	0.167	[−0.045 - 0.365]	0.122	0.438
Employment	Unemployed	-	-	-	-
	Employed	0.110	[−0.103 - 0.313]	0.311	0.699
	Paid leave	−0.199	[−0.393 - 0.012]	0.064	0.438
	Retired	−0.077	[−0.283 - 0.136]	0.479	0.719
Psychological Counseling	No	-	-	-	-
	Yes	0.092	[−0.121 - 0.297]	0.396	0.712
Social support	No	-	-	-	-
	Yes	0.173	[−0.039 - 0.370]	0.110	0.438
Skin Involvement Index*	1 - 3	0.030	[−0.182 - 0.239]	0.781	0.863
HADS	DEP (0 - 21)	−0.153	[−0.352 - 0.059]	0.157	0.470
	ANS (0 - 21)	0.055	[−0.157 - 0.263]	0.611	0.768
EuroQoL	0.00 - 1.00	0.084	[−0.129 - 0.290]	0.439	0.718
EuroQol Visual Scale	0 - 100	0.055	[−0.160 - 0.265]	0.615	0.768
S-BIS modified	0 - 24	0.187	[−0.025 - 0.382]	0.084	0.438
PNRS	0 - 10	−0.051	[−0.260 - 0.162]	0.640	0.768

ρ = Spearman’s correlation coefficient; CI = confidence interval; p significance levels: <0.05 (*), <0.01 (**), <0.001 (***), FDR–BH = false discovery rate corrected *p*-value (Benjamini–Hochberg). HADS = Hospital Anxiety and Depression Scale; S-BIS modified = Spanish Body Image Scale (modified); PNRS = Pain Numerical Rating Scale. The first category of each categorical variable was used as the reference group. *Skin involvement index analyzed as a quantitative variable for correlation

**Table 4:** Correlations between sociodemographic/clinical variables and executive function Z-scores in adults with NF1

Variables	Categories/ Ranges	Executive function			
		Spearman (ρ)	95% CI (ρ)	*p*-value	p (FDR–BH)
Age	16 - 74	−0.023	[−0.233–0.188]	0.830	0.830
Marital Status	Single	-	-	-	-
	Married	−0.208	[−0.401–0.003]	0.054	0.247
	Divorced	−0.103	[−0.307–0.110]	0.342	0.462
Education	0 - 17	0.267	[0.060–0.452]	0.012**	0.110
Employment	Unemployed	-	-	-	-
	Employed	−0.137	[−0.338–0.076]	0.206	0.352
	Paid leave	0.263	[0.056–0.449]	0.014*	0.110
	Retired	−0.083	[−0.289–0.130]	0.444	0.547
Psychological Counseling	No	-	-	-	-
	Yes	0.181	[−0.030–0.378]	0.093	0.247
Social support	No	-	-	-	-
	Yes	0.182	[−0.030–0.378]	0.092	0.247
Skin Involvement Index*	1 - 3	0.140	[−0.072–0.341]	0.195	0.352
HADS	DEP (0 - 21)	−0.133	[−0.334–0.080]	0.220	0.352
	ANS (0 - 21)	−0.065	[−0.272–0.147]	0.547	0.625
EuroQoL	0.00 - 1.00	−0.200	[−0.394–0.011]	0.063	0.247
EuroQol Visual Scale	0 - 100	−0.103	[−0.310–0.112]	0.346	0.462
S-BIS modified	0 - 24	−0.047	[−0.255–0.165]	0.665	0.709
PNRS	0 - 10	0.148	[−0.066–0.349]	0.173	0.352

ρ = Spearman’s correlation coefficient; CI = confidence interval; p significance levels: <0.05 (*), <0.01 (**), <0.001 (***), FDR–BH = false discovery rate corrected *p*-value (Benjamini–Hochberg). HADS = Hospital Anxiety and Depression Scale; S-BIS modified = Spanish Body Image Scale (modified); PNRS = Pain Numerical Rating Scale. The first category of each categorical variable was used as the reference group. *Skin involvement index analyzed as a quantitative variable for correlation

**Table 5:** Correlations between sociodemographic/clinical variables and visuospatial ability Z-scores in adults with NF1

Variables	Categories/ Ranges	Visuospatial			
		Spearman (ρ)	95% CI (ρ)	*p*-value	p (FDR–BH)
Age	16 - 74	−0.025	[−0.237 - 0.189]	0.818	0.981
Marital Status	Single	-	-	-	-
	Married	−0.014	[−0.227 - 0.199]	0.896	0.987
	Divorced	−0.096	[−0.303 - 0.119]	0.380	0.571
Education	0 - 17	0.186	[−0.029 - 0.384]	0.089	0.307
Employment	Unemployed	-	-	-	-
	Employed	0.035	[−0.179 - 0.246]	0.749	0.963
	Paid leave	−0.230	[−0.423 - −0.018]	0.034	0.304
	Retired	−0.136	[−0.339 - 0.080]	0.216	0.389
Psychological Counseling	No	-	-	-	-
	Yes	−0.198	[−0.395 - 0.016]	0.069	0.307
Social support	No	-	-	-	-
	Yes	−0.009	[−0.222 - 0.204]	0.932	0.987
Skin Involvement Index*	1 - 3	<0.001	[−0.213 - 0.214]	0.997	0.997
HADS	DEP (0 - 21)	−0.251	[−0.441 - −0.040]	0.020*	0.304
	ANS (0 - 21)	−0.195	[−0.392 - 0.019]	0.074	0.307
EuroQoL	0.00 - 1.00	0.155	[−0.060 - 0.356]	0.157	0.353
EuroQol Visual Scale	0 - 100	0.099	[−0.119 - 0.308]	0.372	0.571
S-BIS modified	0 - 24	−0.141	[−0.344 - 0.074]	0.198	0.389
PNRS	0 - 10	−0.171	[−0.372 - 0.045]	0.119	0.307

ρ = Spearman’s correlation coefficient; CI = confidence interval; p significance levels: <0.05 (*), <0.01 (**), <0.001 (***), FDR–BH = false discovery rate corrected *p*-value (Benjamini–Hochberg). HADS = Hospital Anxiety and Depression Scale; S-BIS modified = Spanish Body Image Scale (modified); PNRS = Pain Numerical Rating Scale. The first category of each categorical variable was used as the reference group. *Skin involvement index analyzed as a quantitative variable for correlation

**Table 6:** Correlations between sociodemographic/clinical variables and visuomotor speed Z-scores in adults with NF1

Variables	Categories/ Ranges	Visuomotor speed			
		Spearman (ρ)	95% CI (ρ)	*p*-value	p (FDR–BH)
Age	16 - 74	0.235	[0.023 - 0.427]	0.030*	0.273
Marital Status	Single	-	-	-	-
	Married	0.208	[−0.006 - 0.403]	0.056	0.338
	Divorced	−0.096	[−0.303 - 0.119]	0.381	0.955
Education	0 - 17	0.263	[0.052 - 0.450]	0.015*	0.273
Employment	Unemployed	-	-	-	-
	Employed	0.137	[−0.078 - 0.340]	0.210	0.945
	Paid leave	−0.030	[−0.241 - 0.185]	0.788	0.955
	Retired	0.009	[−0.204 - 0.222]	0.932	0.955
Psychological Counseling	No	-	-	-	-
	Yes	−0.038	[−0.249 - 0.177]	0.731	0.955
Social support	No	-	-	-	-
	Yes	−0.123	[−0.327 - 0.093]	0.263	0.946
Skin Involvement Index*	1 - 3	0.006	[−0.207 - 0.219]	0.955	0.955
HADS	DEP (0 - 21)	−0.021	[−0.233 - 0.193]	0.850	0.955
	ANS (0 - 21)	0.041	[−0.174 - 0.252]	0.711	0.955
EuroQoL	0.00 - 1.00	0.041	[−0.173 - 0.252]	0.706	0.955
EuroQol Visual Scale	0 - 100	−0.053	[−0.265 - 0.165]	0.636	0.955
S-BIS modified	0 - 24	0.035	[−0.180 - 0.246]	0.754	0.955
PNRS	0 - 10	0.092	[−0.125 - 0.300]	0.406	0.955

ρ = Spearman’s correlation coefficient; CI = confidence interval; p significance levels: <0.05 (*), <0.01 (**), <0.001 (***), FDR–BH = false discovery rate corrected *p*-value (Benjamini–Hochberg). HADS = Hospital Anxiety and Depression Scale; S-BIS modified = Spanish Body Image Scale (modified); PNRS = Pain Numerical Rating Scale. The first category of each categorical variable was used as the reference group. *Skin involvement index analyzed as a quantitative variable for correlation

The analysis of correlations between visuospatial ability, visuomotor speed, attention, and working memory in adults with NF1 revealed several small to moderate associations with sociodemographic variables at the uncorrected level. Visuomotor speed showed positive correlations with age (ρ = 0.235, 95% CI [0.023–0.427], *p* = 0.030) and years of education (ρ = 0.263, 95% CI [0.052–0.450], *p* = 0.015). Attention showed negative correlations with paid leave status (ρ = −0.274, 95% CI [−0.459 to −0.066], *p* = 0.011), while working memory showed positive correlations with years of education (ρ = 0.212, 95% CI [0.001–0.404], *p* = 0.049) and paid leave status (ρ = 0.220, 95% CI [0.009–0.411], *p* = 0.041). However, none of these associations remained significant after correction for multiple comparisons. Regarding clinical variables, visuospatial ability showed a small negative correlation with depressive symptoms measured using the HADS (ρ = −0.251, 95% CI [−0.441 to −0.040], *p* = 0.020), although this association did not remain significant after correction for multiple comparisons. Body image was not significantly associated with attention (ρ = −0.101, *p* > 0.10), and no significant associations were observed between other clinical outcomes, including pain and quality of life, and the cognitive domains assessed (Tables 5, 6, 7 and Table 8).

**Table 7:** Correlations between sociodemographic/clinical variables and attention Z-scores in adults with NF1

Variables	Categories/ Ranges	Attention			
		Spearman (ρ)	95% CI (ρ)	*p*-value	p (FDR–BH)
Age	16 - 74	0.045	[−0.168 - 0.255]	0.678	0.987
Marital Status	Single	-	-	-	-
	Married	0.071	[−0.143 - 0.279]	0.516	0.929
	Divorced	0.150	[−0.064 - 0.351]	0.168	0.583
Education	0 - 17	0.206	[−0.007 - 0.400]	0.058	0.346
Employment	Unemployed	-	-	-	-
	Employed	0.141	[−0.073 - 0.343]	0.194	0.583
	Paid leave	−0.274	[−0.459 - −0.066]	0.011*	0.193
	Retired	0.100	[−0.114 - 0.306]	0.359	0.717
Psychological Counseling	No	-	-	-	-
	Yes	−0.010	[−0.221 - 0.202]	0.927	0.987
Social support	No	-	-	-	-
	Yes	0.005	[−0.207 - 0.216]	0.966	0.987
Skin Involvement Index*	1 - 3	−0.214	[−0.408 - −0.002]	0.048*	0.346
HADS	DEP (0 - 21)	0.017	[−0.195 - 0.228]	0.876	0.987
	ANS (0 - 21)	0.012	[−0.200 - 0.224]	0.910	0.987
EuroQoL	0.00 - 1.00	−0.002	[−0.214 - 0.210]	0.987	0.987
EuroQol Visual Scale	0 - 100	0.008	[−0.207 - 0.222]	0.941	0.987
S-BIS modified	0 - 24	−0.101	[−0.306 - 0.113]	0.355	0.717
PNRS	0 - 10	−0.124	[−0.328 - 0.092]	0.259	0.665

ρ = Spearman’s correlation coefficient; CI = confidence interval; p significance levels: <0.05 (*), <0.01 (**), <0.001 (***), FDR–BH = false discovery rate corrected *p*-value (Benjamini–Hochberg). HADS = Hospital Anxiety and Depression Scale; S-BIS modified = Spanish Body Image Scale (modified); PNRS = Pain Numerical Rating Scale. The first category of each categorical variable was used as the reference group. *Skin involvement index analyzed as a quantitative variable for correlation

**Table 8:** Correlations between sociodemographic/clinical variables and working memory Z-scores in adults with NF1

Variables	Categories/ Ranges	Working memory			
		Spearman (ρ)	95% CI (ρ)	*p*-value	p (FDR–BH)
Age	16 - 74	0.004	[−0.207 - 0.214]	0.973	0.973
Marital Status	Single	-	-	-	-
	Married	−0.026	[−0.236 - 0.185]	0.809	0.973
	Divorced	0.083	[−0.130 - 0.288]	0.446	0.973
Education	0 - 17	0.212	[0.001 - 0.404]	0.049*	0.441
Employment	Unemployed	-	-	-	-
	Employed	−0.140	[−0.341 - 0.072]	0.194	0.792
	Paid leave	0.220	[0.009 - 0.411]	0.041*	0.441
	Retired	−0.017	[−0.227 - 0.194]	0.873	0.973
Psychological Counseling	No	-	-	-	-
	Yes	0.030	[−0.181 - 0.240]	0.780	0.973
Social support	No	-	-	-	-
	Yes	0.006	[−0.205 - 0.216]	0.959	0.973
Skin Involvement Index*	1 - 3	0.074	[−0.139 - 0.281]	0.494	0.973
HADS	DEP (0 - 21)	−0.007	[−0.217 - 0.204]	0.951	0.973
	ANS (0 - 21)	0.133	[−0.080 - 0.334]	0.220	0.792
EuroQoL	0.00 - 1.00	0.038	[−0.174 - 0.247]	0.723	0.973
EuroQol Visual Scale	0 - 100	−0.160	[−0.361 - 0.055]	0.144	0.792
S-BIS modified	0 - 24	−0.021	[−0.230 - 0.191]	0.849	0.973
PNRS	0 - 10	0.045	[−0.168 - 0.254]	0.680	0.973

ρ = Spearman’s correlation coefficient; CI = confidence interval; p significance levels: <0.05 (*), <0.01 (**), <0.001 (***), FDR–BH = false discovery rate corrected *p*-value (Benjamini–Hochberg). HADS = Hospital Anxiety and Depression Scale; S-BIS modified = Spanish Body Image Scale (modified); PNRS = Pain Numerical Rating Scale. The first category of each categorical variable was used as the reference group. *Skin involvement index analyzed as a quantitative variable for correlation

## Discussion

This research explored cognitive performance of NF1 adults, it also assessed the association between cognitive performance and clinical outcomes like depression symptoms and skin involvement.

Our participants’ results retrieved low skin severity index, with good QoL, low body image related concerns, no symptoms of depression nor anxiety and low level of feeling pain. Despite this, NF1 patients exhibited cognitive underperformance. They performed below the normative mean across several cognitive domains relative to age-adjusted Spanish reference data. This pattern was particularly evident in attention, with mean scores approximately one standard deviation below the normative mean (z = −1.171), within the low-average range, consistent with recent population-based findings in adults with NF1 [19]. Of the seven evaluated domains, executive function showed performance more than two standard deviations below the normative mean, indicating clinically significant impairment [60].

Together with recent population-based and neuropsychological studies, our results support the interpretation that cognitive functioning in NF1 adulthood is associated with a pattern of domain-specific neurocognitive vulnerability rather than generalized cognitive decline [19, 20].

It is worth noting that both attention and executive functions, along with alterations in processing speed, were linked to dysfunctions in front-subcortical brain circuits [61, 62]. These findings supported the notion that cognitive impairments in NF1 are intrinsic to the condition, likely due to genetic factors and/or dysfunctional neural pathways [6, 8]. Specifically, results of this research were consistent with previous studies showing that individuals with NF1 exhibit reduced activation in brain regions essential for attention and memory, including the left dorsolateral prefrontal cortex (DLPFC) and right intraparietal sulcus (IPS). Additionally, compared to healthy controls, they show less deactivation of the posterior cingulate cortex (PCC) and altered connectivity between the PCC and regions of the default mode network (DMN), including visual, cerebellar, and temporal areas. This aberrant functional pattern, marked by under-recruitment of task-relevant areas and insufficient suppression of task-irrelevant ones, might have disrupted working memory performance in NF1 [63, 64].

Impairments in these areas could negatively affect the well-being of these patients and make daily life more challenging. Executive function deficits, such as diminished inhibitory control and increased reliance on external stimuli to stay focused, were linked to poorer reasoning and working memory performance, potentially interfering with cognitive control [63]. Recent population-based studies in adults with NF1 have reported impairments in executive functioning that are consistent with the executive difficulties observed in our sample. However, these studies also showed that some executive domains, such as working memory, remained preserved despite participants reporting difficulties in this area in everyday life [19]. In our sample, working memory performance did not reach clinically significant impairment, although scores were slightly below normative expectations. This pattern suggests that relative underperformance in working memory may be associated with the subjective perception of reduced cognitive efficiency in daily life compared with the general population. Notably, this relative underperformance was not associated with reduced quality of life in our sample, possibly because our study assessed objective cognitive performance rather than participants’ perceptions of their everyday executive functioning. Executive dysfunction observed in the present study may also help explain previously reported alterations in social cognition in adults with NF1, supporting the hypothesis that executive vulnerability may be associated with previously described difficulties in social cognitive functioning in this population [20].

Attention problems, even when not related to ADHD, were associated with learning and mental health difficulties [65]. Sustained attention demands might have led to fatigue and reduced coping capacity, which is linked to daily functioning [22, 66]. These challenges can also affect social skills and limit access to employment, reducing autonomy and integration.

To explore whether cognitive impairments in adults with NF1 were associated with psychological or physical clinical variables, we examined the relationships between cognitive performance and the clinical outcomes assessed in the study. Neither the GCI nor the individual cognitive domains showed significant associations with the clinical variables evaluated after correction for multiple comparisons, supporting the interpretation that cognitive alterations observed in this population may reflect intrinsic neurocognitive characteristics of NF1 rather than secondary effects related to psychological distress or disease burden [19, 20]. Nevertheless, domain-level analyses revealed small associations with two specific clinical outcomes prior to correction for multiple comparisons. Although these associations did not remain significant after correction, they are worth reporting as they may suggest subtle relationships between specific clinical features and specific cognitive domains.

Visuospatial ability appeared particularly sensitive to depression in this population. Other studies have indicated that, in pediatric NF1 cases, depressive symptoms are linked to declines in visuospatial skills [28, 67]. In our sample, patients were excluded if they presented depression; however, they still exhibited difficulties in visuospatial ability, suggesting that these impairments are more likely related to underlying cognitive characteristics of NF1 rather than emotional factors. This interpretation is further supported by recent population-based evidence reporting impairments in immediate visuospatial recall in adults with NF1 [19].

Similarly, attentional performance showed a small association with the NF1 cutaneous severity index with individuals presenting higher levels of skin severity tending to perform worse in attention tasks. However, most participants in our sample were classified within grade 1 severity and already showed attentional difficulties, indicating that these alterations cannot be explained solely by disease severity [19, 30]. Consistent with previous findings, clinical severity does not appear to represent a robust predictor of cognitive functioning in adults with NF1 [20].

Both attention and visuospatial processes are associated with the frontal and parietal lobes, which have previously been shown to exhibit reduced activation in individuals with NF1 [63, 64]. Moreover, these regions were likewise involved in mechanisms related to depression and body awareness [68, 69]. Although body image in our sample did not show significant associations with these cognitive domains, previous studies have shown that greater visibility of skin manifestations in NF1 is associated with increased body image concerns and psychosocial burden, which may increase cognitive load during assessment situations and selectively interfere with attentional performance [32, 40]. Establishing a clear causal link between cognitive impairments and their psychological correlates remains challenging; however, our results suggest that attentional and visuospatial difficulties are present independently of disease severity and depressive symptomatology, although more pronounced skin manifestations and higher levels of depressive symptoms may be associated with poorer performance in these domains.

Educational attainment emerged as the main sociodemographic factor associated with the Global Cognitive Index in this study and may reflect the role of cognitive reserve in adults with NF1. Although NF1-related cognitive difficulties are typically neurodevelopmental and arise early in life, higher educational attainment may support compensatory strategies that help maintain cognitive performance within the normal range despite underlying vulnerabilities [70]. A small association between age and visuomotor performance was also observed; however, this finding did not remain significant after correction for multiple comparisons and was not replicated across other cognitive domains. Therefore, it is unlikely to represent a meaningful age-related effect and may instead reflect sample-specific variability.

This study has several methodological strengths. It focused on adults with NF1 without neurological or psychiatric comorbidities, allowing a clearer identification of cognitive alterations intrinsic to NF1 rather than secondary to associated conditions. This improves the precision of the neurocognitive profile compared with previous studies using more heterogeneous samples. Additionally, the study used standardized neuropsychological measures across multiple cognitive domains and included relevant clinical variables—such as depressive symptoms, skin involvement, pain, quality of life, and body image providing a comprehensive assessment of factors influencing cognitive performance.

Some limitations should be considered when interpreting these findings. Participants with clinically significant depressive symptoms were excluded to reduce psychological confounding and better isolate NF1-related cognitive alterations, which strengthened internal validity but may limit ecological validity and generalizability to the broader NF1 population, especially those with higher psychological burden. Another limitation concerns the use of the GCI as a descriptive composite measure rather than a psychometrically validated indicator of general cognitive ability, meaning it should be interpreted cautiously. However, domain-specific analyses were also conducted to support a more precise neuropsychological interpretation. Similar composite indices have been employed in previous studies as summary measures of global cognitive performance across multiple domains [71]. Methodologically, the cross-sectional design prevents causal conclusions. Additionally, recruitment from a single national reference center, reliance on Spanish normative data instead of a matched control group, and the absence of neuroimaging data further limit generalizability and the ability to directly link cognitive findings to underlying neural mechanisms.

Future research on adults with NF1 should focus especially on attention and executive functioning to better understand the involvement of front-subcortical brain circuits in cognitive performance. Studies should also examine how different clinical phenotypes are associated with cognitive vulnerability and expand assessments to include social cognition (e.g., emotion recognition, theory of mind, moral reasoning, and social information processing), since these domains affect everyday social functioning. Researchers should investigate the influence of cognitive reserve and subjective cognitive perception on well-being, as they may play an important role even when objective cognitive performance appears within normative ranges. Finally, longitudinal studies with more diverse samples are needed to track cognitive changes over time and clarify how emotional factors interact with NF1-related cognition, helping improve targeted cognitive and psychosocial interventions.

## Conclusions

While the cognitive impairments observed align with previous literature, this study provides additional evidence from individuals with NF1 without other cognitive-related diagnoses, suggesting that cognitive vulnerability may represent a core feature of the condition. These findings. enhance the understanding of NF1-related cognitive difficulties and support further research into rare genetic disorders.

Although clinical factors such as depression and the severity of skin manifestations were associated with specific domains such as visuospatial ability and attention, they did not explain overall cognitive performance. These findings highlight the potential association of cognitive difficulties and daily functioning and support the relevance of targeted cognitive and psychosocial interventions in this population.

Healthcare professionals may consider assessing cognitive functioning in adults with NF1, particularly when patients report cognitive concerns or functional difficulties, in order to better tailor support strategies. Effective professional–patient communication and integration of these aspects into genetic counseling may contribute to improved patient care. Interventions addressing learning difficulties, fatigue, attention, executive functioning, and psychological well-being may be beneficial depending on individual clinical needs. Our findings also highlight the potential importance of educational support and long-term cognitive stimulation in promoting adaptive functioning in adulthood among individuals with NF1.

## Electronic supplementary material

Below is the link to the electronic supplementary material.

Supplementary Material 1

Supplementary Material 2

## Data Availability

The data that support the findings of this study are available from Germans Trias i Pujol Hospital, but restrictions apply to the availability of these data, which were used under license for the current study and are not publicly available. However, the data are available from the authors upon reasonable request and with the permission of Germans Trias I Pujol Hospital.

## Abbreviations

NF1: Neurofibromatosis type 1
CALM’s: Café-au-lait macules
SNF: Spinal Neurofibromatosis
MPNST: Malignant Peripheral Sheath Tumor
PN: Plexiform Neurofibroma
GABA: γ-aminobutyric acid
QoL: Quality of Life
BI: Body Image
CSUR: Centers, Services and Units of Reference
CEIC: Ethics Research Committee
STROBE: Strengthening the Reporting of Observational Studies in Epidemiology
GCI: Global Cognitive Index
HADS: Hospital Anxiety and Depression Scale
S-BIS: Spanish Body Image Scale
PNRS: Pain Numerical Rating Scale
EQ-VAS: EuroQoL Quality of Life Visual Analog Scale
NART: National Adult reading test
WMS: Weschler Memory Scale
WAIS: Weschler Adult Intelligence Scale
TMT: Trail Making Test
CTT: Color Trail Making Test
JLO: Judgement of Line Orientation
SD: Standard Deviation
DLPFC: Left Dorsolateral Prefrontal Cortex
IPS: Right Intraparietal Sulcus
PCC: Posterior Cingulate Cortex
DMN: Default Mode Network
ADHD: Attention Deficit/Hyperactivity Disorder
