# Supplementary material for: Cognitive function in adult NF1 patients without other cognitive disorders: a cross-sectional study
Source: Orphanet J Rare Dis. 2026 Jul 3;21:334. doi: 10.1186/s13023-026-04456-6 (PMC13637243; doi:10.1186/s13023-026-04456-6)
Supplement: Supplementary file 1 [file 13023_2026_4456_MOESM1_ESM.docx]

**Additional File 2:** Internal consistency for the HADS Anxiety and Depression subscales (Cronbach’s α)

| **Variable** | **Subscales** | **Cronbach’s α** |
| --- | --- | --- |
| HADS | ANS | 0.83 |
|  | DEP | 0.61 |

HADS-ANS = Hospital Anxiety and Depression Scale–Anxiety subscale; HADS-DEP = Hospital Anxiety and Depression Scale–Depression subscale; α = Cronbach’s alpha.
