# Supplementary material for: Cognitive function in adult NF1 patients without other cognitive disorders: a cross-sectional study
Source: Orphanet J Rare Dis. 2026 Jul 3;21:334. doi: 10.1186/s13023-026-04456-6 (PMC13637243; doi:10.1186/s13023-026-04456-6)
Supplement: Supplementary file 2 [file 13023_2026_4456_MOESM2_ESM.docx]

| Variables | W statistic | p-value | Normal distribution |
| --- | --- | --- | --- |
| Age | 0.913 | < 0.001 | No |
| Education | 0.860 | < 0.001 | No |
| Skin Involvement Index | 0.738 | < 0.001 | No |
| HADS DEP | 0.912 | < 0.001 | No |
| HADS ANS | 0.969 | 0.032 | No |
| EuroQoL | 0.797 | < 0.001 | No |
| EuroQol Visual Scale | 0.905 | < 0.001 | No |
| S-BIS modified | 0.799 | < 0.001 | No |
| PNRS | 0.798 | < 0.001 | No |
| WMS-IV: Visual reproduction I (Z-score) | 0.969 | 0.033 | No |
| WMS-IV: Visual reproduction II (Z-score) | 0.969 | 0.034 | No |
| WMS-IV: Logical memory I (Z-score) | 0.975 | 0.088 | Yes |
| WMS-IV: Logical memory II (Z-score) | 0.966 | 0.020 | No |
| TMT-A (Z-score) | 0.958 | 0.006 | No |
| TMT-B (Z-score) | 0.928 | < 0.001 | No |
| CTT- 1 (Z-score) | 0.750 | < 0.001 | No |
| CTT- 2 (Z-score) | 0.750 | < 0.001 | No |
| JLO (Z-score) | 0.896 | < 0.001 | No |
| WAIS-IV: Symbols (Z-score) | 0.986 | 0.508 | Yes |
| WAIS-IV: Digit Span backward (Z-score) | 0.941 | < 0.001 | No |
| WAIS-IV: Digit Span forward (Z-score) | 0.959 | 0.008 | No |
| GCI | 0.965 | 0.020 | No |

**Additional File 2:** Results of Shapiro–Wilk normality tests for cognitive and clinical variables included in the statistical analyses

HADS-A = Hospital Anxiety and Depression Scale–Anxiety subscale; HADS-D = Hospital Anxiety and Depression Scale–Depression subscale; EQ-VAS = EuroQol Visual Analogue Scale; PNRS = Pain Numerical Rating Scale; S-BIS modified = Spanish Body Image Scale (modified); WMS-IV = Wechsler Memory Scale–Fourth Edition; WAIS-IV = Wechsler Adult Intelligence Scale–Fourth Edition; GCI = Global Cognitive Index. Normality of continuous variables was assessed using the Shapiro–Wilk test. Normal distribution was assumed when p > 0.05. Results were used to determine the selection of parametric or non-parametric statistical procedures in subsequent analyses.
